# Formation of a highly dense tetra-rhenium cluster in a protein crystal and its implications in medical imaging

**DOI:** 10.1107/S2052252519006651

**Published:** 2019-06-13

**Authors:** Alice Brink, John R. Helliwell

**Affiliations:** a Department of Chemistry, University of the Free State, Nelson Mandela Drive, Bloemfontein, 9301, South Africa; b School of Chemistry, University of Manchester, Brunswick Street, Manchester M13 9PL, UK

**Keywords:** rhenium tetranuclear clusters, thera­nostics, time-resolved protein crystallography, chemical crystallography, medical imaging

## Abstract

The fact that a protein crystal can serve as a chemical reaction vessel is intrinsically fascinating. That it can produce an electron-dense tetranuclear rhenium cluster compound from a rhenium tri­carbonyl tri­bromo starting compound adds to the fascination. Such a cluster has been synthesized previously *in vitro*, where it formed under basic conditions. Therefore, its synthesis in a protein crystal grown at pH 4.5 is even more unexpected. The X-ray crystal structures presented here are for the protein hen egg-white lysozyme incubated with a rhenium tri­carbonyl tri­bromo compound for periods of one and two years. These reveal a completed, very well resolved, tetra-rhenium cluster after two years and an intermediate state, where the carbonyl ligands to the rhenium cluster are not yet clearly resolved, after one year. A dense tetranuclear rhenium cluster, and its technetium form, offer enhanced contrast in medical imaging. Stimulated by these crystallography results, the unusual formation of such a species directly in an *in vivo* situation has been considered. It offers a new option for medical imaging compounds, particularly when considering the application of the pre-formed tetranuclear cluster, suggesting that it may be suitable for medical diagnosis because of its stability, preference of formation and biological compatibility.

## Abstract 2

Exploration of a ‘time on shelf’ protein structure containing the radiopharmaceutical synthon *fac*-[Re(CO)_3_(H_2_O)_3_]^+^ as an *in vivo* reaction vessel to form tetranuclear rhenium clusters appropriate for theranostic applications is reported.

## 1. Introduction   

The organometallic chemistry of the manganese group 7 triad involving the technetium-99m synthon continues to attract significant attention in the development of modern radiopharmaceuticals. ^99m^Tc remains an important radionuclide for diagnostic nuclear medicine with ∼80% of current radiopharmaceuticals administered clinically containing this radioisotope (Liu, 2004 ▸; Kluba & Mindt, 2013 ▸). The rhenium homolog is regularly utilized as a model for the technetium complex of interest to confirm its coordination and structure, with the advantages that accompany working with a non-radioactive complex (Bordoloi *et al.*, 2015 ▸; Nayak *et al.*, 2013 ▸, 2015 ▸). However, the medical advantages of rhenium should not be underestimated when considering the radionuclides of ^186^Re and ^188^Re. ^186^Re (half life, *t*_1/2_ = 3.7 days; maximum tissue penetration depth = 5 mm) and ^188^Re (*t*_1/2_ = 17 h; maximum tissue penetration depth = 11 mm) contain both β- and γ-emission allowing for therapeutic treatment with simultaneous imaging potential (Bowen & Orvig, 2008 ▸; Volkert & Hoffman, 1999 ▸; Dilworth & Parrott, 1998 ▸). The possibility of combining rhenium and technetium into a single multifunctional agent to be used simultaneously for imaging and therapy has potential for the development of theranostic agents (Svenson, 2013 ▸; Alberto, 2018 ▸; Spagnul *et al.*, 2013 ▸). The success of ‘target-specific’ radiopharmaceutical development relies on the targeting biomolecule (linked to a radionuclide) coordinating to the bio-receptors with high affinity and specificity, and compounds have to date been primarily mononuclear, *i.e.* one metal centre, in form (Liu, 2004 ▸). The potential of multifunctional agents leads to increased strategies for exploiting multi-nuclear complexes as illustrated by the increased cytotoxicity of dinuclear, pyridine-linked, rhenium(I) tri­carbonyl complexes in comparison with their mononuclear counterparts (Ye *et al.*, 2016 ▸). Similarly examples of dinuclear high-valent rhenium (V and III) complexes exist with anti-cancer activity (Konkankit *et al.*, 2018 ▸). The complexity of the synthesis of multi-nuclear complexes has hindered interest in medical use. However, the reactivity studies on manganese(I) tri­carbonyl complexes have provided insight into the manner in which the nuclearities of rhenium(I) tri­carbonyl complexes can be manipulated to form either mono- or dinuclear species (Mokolokolo *et al.*, 2018 ▸). This has been followed by two strategies which can prepare multinuclear rhenium(I) and technetium(I) tri­carbonyl complexes, either di- or tetranuclear clusters, in a one-pot reaction. Furthermore, it allows for a tetranuclear cluster containing both rhenium and technetium metal centres to be a model theranostic agent (Frei *et al.*, 2018 ▸).

Our interest is in understanding the interactions between organometallic complexes with proteins in a similar manner to that described by the fragment-based drug-design method (Joseph-McCarthy *et al.*, 2014 ▸; Erlanson, 2012 ▸; Murray *et al.*, 2012 ▸), whereby protein-ligand binding of low molecular weight fragments, either as organic or organometallic precursors, can be exploited to derive a model for radiopharmaceutical lead compounds. Protein crystallography studies reporting rhenium coordination continue to be rare and tend to report binding preference to histidine imidazole (Binkley *et al.*, 2011 ▸; Zobi & Spingler, 2012 ▸; Santoro *et al.*, 2012 ▸; Takematsu *et al.*, 2013 ▸), with the exception of our study, which employed two-X-ray-wavelength enhancement and discrimination of rhenium, even at low occupancy, and whereby rhenium was observed to bind to aspartic acid, glutamic acid, arginine and leucine residues as well as to histidine (Brink & Helliwell, 2017 ▸). We hereby extend the understanding of organometallic complex interactions with proteins by reporting here one- and two-year time-on-the-shelf crystal structures of hen egg-white lysozyme (HEWL), *i.e.* incubated with the rhenium tri­carbonyl tri­bromo compound over a period of 24 months. The structures reveal a completed, very well resolved, tetranuclear rhenium(I) tri­carbonyl cluster after two years and an intermediate state after one year.

## 2. Experimental   

### 2.1. Crystallization   

Standard HEWL (20 mg) crystallization conditions were used consisting of 10% NaCl, 0.04 *M* sodium acetate (pH 4.7) and *fac*-[Et_4_N]_2_[Re(CO)_3_(Br)_3_] at 0.03 *M* in 1.4 ml water, in sitting-drop conditions, initially included with dimethyl sulfoxide (DMSO) [at 7.5%(*v*/*v*)]. Pure silicone oil was used as a cryoprotectant and yielded consistent and good diffraction since use of Paratone oil tended to cause decomposition of the crystals upon contact. The crystal was transferred into the oil on a microscope slide and moved to allow complete coating for ∼3 s. Crystals from identical trays grew over a period of approximately three weeks. Diffraction data were measured after three weeks and reported by Brink & Helliwell (2017 ▸). Crystals in individual trays were left undisturbed for a period of one (1Yr-Y) and two years (2Yr-X) at ∼25°C, after which time they were harvested and X-ray diffraction data measured and analysed individually.

### 2.2. Infrared spectroscopy and kinetic principles of cluster formation   

To evaluate whether the rhenium tetranuclear cluster formation could be induced by the presence of the protein or under the specific buffer solution, the following infrared (IR) study was conducted (see Fig. S2 in the Supporting information). Pure crystalline product of the structures reported here (after two years retained in the sitting drop) was analysed by IR [crystalline solid state, attenuated total reflectance (ATR), cm^−1^]: *v*_(CO)_ = 2023, 1907. The pure rhenium tetranuclear cluster complex [Re_4_(μ_3_-OH)_4_(CO)_12_], synthesized according to Egli *et al.* (1997 ▸), indicated near identical carbonyl stretching frequencies within a margin of error. IR (solid state, ATR, cm^−1^): *v*_(CO)_ = 2028, 1913, 1885. The starting mononuclear rhenium complex, *fac*-[Et_4_N]_2_[Re(CO)_3_(Br)_3_], was then dissolved in identical buffer conditions (10% NaCl, 0.04 *M* sodium acetate, pH 4.7) without the addition of the protein for a three-month period and did not yet indicate rhenium tetranuclear cluster formation. IR (solid state, ATR, cm^−1^): *v*_(CO)_ = 2016, 1870. For the sake of comparison, the IR of the pure starting mononuclear rhenium complex, *fac*-[Et_4_N]_2_[Re(CO)_3_(Br)_3_] is as follows, IR (solid state, ATR, cm^−1^): *v*_(CO)_ = 1996, 1847. This suggests that the formation of the tetranuclear cluster in these structures involves a complex interplay of factors, which includes the presence of the protein and not only the salt buffer conditions.

Metal clusters (or multinuclear complexes) are rarely considered as viable options in medicinal inorganic chemistry, primarily due to the complexity of the synthesis. Our research has indicated that not only can cluster complexes hold mixed metals, applicable for theranostic (simultaneous therapy and diagnosis) (Frei *et al.*, 2018 ▸) applications, but also that time is a factor in the formation of the clusters (Mokolokolo *et al.*, 2018 ▸). The formation of multinuclear Re/Tc complexes, for example dinuclear (Re_2_), trinuclear (Re_3_) and tetranuclear (Re_4_) from the mononuclear rhenium complex, {Re_1_ = *fac*-[Et_4_N]_2_[Re(CO)_3_(Br)_3_]}, is proposed to have kinetic rates of second order (Re_2_), third order (Re_3_) and fourth order (Re_4_), respectively, and therefore absolute metal concentration is critically important for the observed rate of formation. This can be manipulated under radiopharmaceutical conditions by increasing the metal concentration and therefore speeding up the formation reaction significantly. For example, from basic kinetic principles assuming dependence on different orders, *i.e.* the formation rate of the tetranuclear cluster will approximate to the fourth order, depending on which steps are actually rate determining. Based on the conditions used during crystallization in this article and a half life of around one year under the conditions studied (Re_1_ concentration = 30 m*M*) the *t*_1/2_ can be reduced to less than a day (∼7 h) by increasing the rhenium concentration by one order of magnitude. Although not necessarily viable under protein crystallization conditions due to protein precipitation, in principle it is accessible under pre-selected radiopharmaceutical conditions. Considering the half life of the radionuclides ^186^Re (*t*_1/2_ = 3.7 d) and ^188^Re (*t*_1/2_ = 17 h) this renders the tetranuclear cluster clearly accessible simply by metal concentration manipulation.

### 2.3. X-ray data collection, structure solution and refinement   

X-ray diffraction data were collected on Beamline I04 at Diamond, with an X-ray wavelength of 0.9763 Å so as to optimize the rhenium *f*′′ anomalous signal at the rhenium *L*_I_ absorption edge. The 1Yr-Y and 2Yr-X X-ray diffraction data collections were carried out on runs one year apart at a fixed temperature of 100 K for the samples. Data and space-group validation were further confirmed with *Zanuda* and *Mosflm* (Leslie & Powell, 2007 ▸; Battye *et al.*, 2011 ▸) in the *CCP*4 software suite. The Diamond automatic processing was utilized, taking the *Xia2* program mtz file. We have also made extensive use of the Cambridge Structural Database (CSD; Allen, 2002 ▸) by using the rigorous search tools that the CSD provides.

The protein crystal structures were solved via molecular replacement using the reported lysozyme structure (PDB entry 2w1y; Cianci *et al.*, 2008 ▸) as a molecular search model within *Phaser* (McCoy *et al.*, 2007 ▸) and then refined in *REFMAC5* (Vagin & Teplyakov, 2010 ▸) in *CCP*4*i* (Potterton *et al.*, 2018 ▸). Space-group validation was considered in triclinic (*P*1), orthorhombic (*P*2_1_2_1_2_1_) and tetragonal (*P*4_3_2_1_2) space groups utilizing *Zanuda* and *Mosflm* in the *CCP*4 software suite (Potterton *et al.*, 2018 ▸; Lebedev & Isupov, 2014 ▸; Leslie & Powell, 2007 ▸).

Model building and adjustment were conducted within the *Coot* molecular graphics program (Emsley & Cowtan, 2004 ▸) alternated with cycles of *REFMAC5*, respectively, in *CCP*4*i*. Furthermore, as the results described below showed unusually electron-dense metal clusters, model refinement for a software comparison was also conducted in *PHENIX* (Afonine *et al.*, 2012 ▸). The metal ligand-binding occupancies were initially calculated using *SHELXTL* (Sheldrick, 2015 ▸) with further manual adjustment guided by residual *F*_o_ − *F*_c_ electron-density peak evidence. Ligand CIF files (RRE, KBW and QEB) were determined from small-molecule crystal structure data sets and then refined by *PHENIX ReadySet*, *eLBOW* (Moriarty *et al.*, 2009 ▸) and *REEL* software (Moriarty *et al.*, 2017 ▸). In the 2Yr-X structure, the *R* and *R*_free_ are quite close in value, 11.17 versus 11.81% *i.e* a gap of just 0.6% rather than the typical 3%. A random atom shift in order to reassert the independence of the *R*_free_ was conducted using *PHENIX Simple Dynamics*. However, no improvement to the difference value of *R*/*R*_free_ occurred and the gap remained at ∼0.6%; a possible cause may be the high metal content of the complex which does prove to be a challenge to the protein-refinement software programs. As mentioned above, we did therefore conduct model refinement utilizing *SHELX*, *CCP*4*i* and *PHENIX* and found, in general, that the *PHENIX* refinement coped best with the high metal electron-density concentration.

The quasi bite angle, in addition to the specific bond distances measured [and supported by the diffraction precision index (DPI); Gurusaran *et al.*, 2014 ▸; Kumar *et al.*, 2015 ▸], is a parameter which was used extensively during this study. It is defined here as the angle formed between the rhenium metal and cognate amino-acid-residue atoms, which gives increased insight into the binding mode compared with small-molecule rhenium bite angles and related bond distances.

The PDB deposition codes for the 1Yr-Y and 2Yr-X structures are 6ro5 and 6ro3, respectively. The raw diffraction images are available in the Zenodo repository (https://doi.org/10.5281/zenodo.2874342). Table 1 ▸ provides a summary of the diffraction data and the model refinements.

## 3. Results and discussion   

Prolonged chemical exposure, alternatively described as ‘time on shelf’ studies have provided valuable insight into protein interactions with organometallic complexes. The anticancer complexes of cisplatin and carboplatin have been reported to coordinate to the N^δ^ and N^∊^ atoms of the His15 residue in HEWL, only in the presence of DMSO. In aqueous conditions, no platinum coordination to the His15 residue is observed after four days of crystallization and growth, indicating that DMSO is able to promote the coordination to the histidine residue in a manner currently not understood (Tanley *et al.*, 2012*b* ▸). However, prolonged chemical exposure of cisplatin, over a period of 15 months, resulted in binding to HEWL in the absence of DMSO (Tanley *et al.*, 2012*a* ▸) – a factor which should be considered for patients who may experience prolonged chemotherapeutic treatment.

In the field of radiopharmaceutical drug development, time and reaction rates obviously play a role, as radioactive half life must be considered. The coordination of N,O bidentate ligands with rhenium tri­carbonyl complexes have, to date, consistently tended to form mononuclear species (Mundwiler *et al.*, 2004 ▸; Schutte *et al.*, 2011 ▸; Brink *et al.*, 2014 ▸). Studies involving the manganese and technetium chemical congener have recently indicated that the nuclearity of the rhenium metal complexes, *i.e.* mononuclear versus dinuclear species, can be manipulated (Mokolokolo *et al.*, 2018 ▸). The application of tetranuclear complexes also creates a window of opportunity for the development of mixed rhenium–technetium tetranuclear clusters for the development of theranostic agents (Frei *et al.*, 2018 ▸), previously less explored due to the complexity of their synthesis.

**Figure 1:**
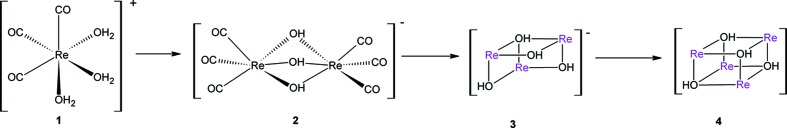
Formation of the dinuclear, trinuclear and tetranuclear rhenium clusters from *fac*-[Re(CO)_3_(H_2_O)_3_]^+^. Note Re, as indicated in complexes 3 and 4 represents the [Re(CO)_3_] fragment. Illustrated without the carbonyl ligands for the sake of clarity.

The various multinuclear cluster formations of rhenium tri­carbonyl complexes in aqueous medium, as a function of pH, have been described in detail by Egli *et al.* (1997 ▸) and Alberto *et al.* (1999 ▸) to show a complex network of species (Fig. 1 ▸). In general, the starting synthon of the rhenium tri­carbonyl complexes, *fac*-[NEt_4_]_2_[Re(CO)_3_(Br)_3_] readily substitutes the bromido atoms in aqueous solutions to form *fac*-[Re(CO)_3_(H_2_O)_3_]^+^. The p*K*_a_ value of the *fac*-[Re(CO)_3_(H_2_O)_3_]^+^ is 7.5 and is predominant under acidic conditions, whereas the trinuclear species [Re_3_(CO)_9_(μ_2_-OH)_3_(μ_3_-OH)]^−^ is predominant under neutral conditions. The formation of the tetranuclear species [Re_4_(μ_3_-OH)_4_(CO)_12_] can occur stepwise from *fac*-[Re(CO)_3_(H_2_O)_3_]^+^ through [Re_2_(CO)_6_(μ_2_-OH)_3_]^−^ and [Re_3_(CO)_9_(μ_2_-OH)_3_(μ_3_-OH)]^−^ under mildly basic conditions. However, the formation of the tetranuclear species as well as the dinuclear rhenium salicyl­idene species are both noted to be affected by long reaction times as well as the presence of organic solvents, such as ether, aceto­nitrile *etc.*, in a manner which is not yet understood (Frei *et al.*, 2018 ▸; Mokolokolo *et al.*, 2018 ▸).

We report here the formation of tetranuclear rhenium(I) tri­carbonyl clusters in HEWL protein incubated with the starting synthon *fac*-[NEt_4_]_2_[Re(CO)_3_(Br)_3_] over a period of 24 months. The structures reveal a completed, very well resolved, tetranuclear cluster after two years and an intermediate state after one year. Our experimental conditions utilized the advantages of tuneable synchrotron radiation at the Diamond Light Source to optimize the rhenium anomalous dispersion signal to a large value (*f*′′ of 12.1 e) at its *L*_I_ absorption edge with a selected X-ray wavelength of 0.9763 Å. When compared with standard laboratory diffraction studies utilizing Cu *K*α X-ray wavelength (1.5418 Å) the Re *f*′′ is only 5.9 e. This allows us to increase the expected peak height by a multiple of 2.1 by optimizing the Re *f*′′. The wavelength-tuning methodology allows for the identification of both the larger rhenium binding-site occupancies as well as the minor occupied ones.

**Figure 2:**
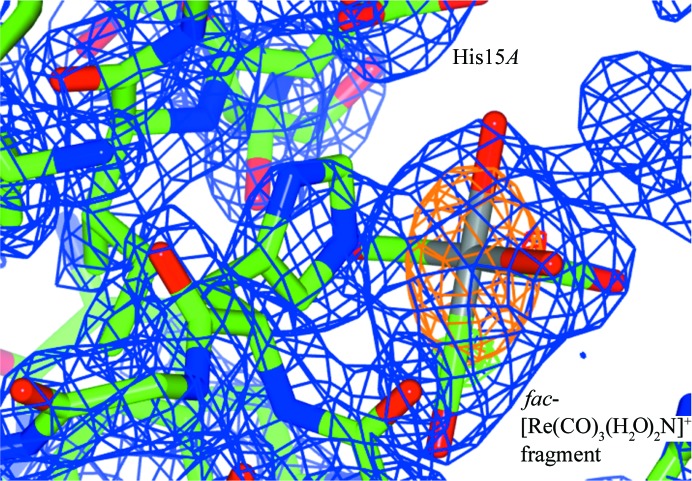
Rhenium mononuclear coordination *fac*-[Re(CO)_3_(H_2_O)_2_N] (N = His15) to His15*A* binding site. Blue shows the 2*F*_o_ − *F*_c_ electron-density map contoured at 1.2 r.m.s., green shows the *F*_o_ − *F*_c_ electron-density map contoured at 5.0σ (the *Coot* default; Emsley & Cowtan, 2004 ▸) and orange shows the anomalous electron-density map contoured at 3.0σ. This figure was prepared using *CCP4mg* (McNicholas *et al.*, 2011 ▸).

The one-year chemical-exposed structure (1Yr-Y) crystallized in the orthorhombic space group *P*2_1_2_1_2_1_ and is refined isotropically due to the data resolution of 1.68 Å. The space-group selection is reminiscent to that previously reported by ourselves (Brink & Helliwell, 2017 ▸) indicating the two protein subunits in the unit cell. We see that rhenium coordination occurs again at the His15*A* residue (Fig. 2 ▸) [Re—N_imidazole_ bond distance = 2.5 (2) Å, occupancy of 80%] and at His15*B* [Re—N_imidazole_ bond distance = 2.6 (2) Å, occupancy of 75%].

Rhenium coordination occurs at Leu129*B* [Re—O = 2.1 (2) Å; occupancy of 55%]. Two rhenium atoms (occupancy 26 and 27%) occur in the vicinity of Glu7*A* and Lys1*A* with Re⋯Re distance of 3.2 (2) Å which is within range of formal interactions as typical Re⋯Re cluster distances are 3.46 and 3.40 Å, while Re⋯Re van der Waals interactions are less than 4.3 Å.

A rhenium atom occurs near Glu35*A* [Re–OE1 = 2.9 (2) Å; occupancy of 20%] as well as between Arg125*A* [Re–NH2 = 2.5 (2) Å; occupancy of 30%] and Asp119*A* [Re–OD2 = 2.4 (2) Å]

A rhenium atom occurs at Asp18*A* [Re–OD1 = 2.2 (2) Å; occupancy of 35%] with sufficient 2*F*_o_ − *F*_c_ density to assign two coordinated aqua ligands.

A rhenium atom occurs at Asp52*A* [Re–OD2 = 2.1 (2) Å; occupancy of 21%] and at Asn46*A* [Re⋯ND2 = 2.9 (2) Å] which is within range of a van der Waals interaction (Re⋯N = 3.7 Å; Re⋯O = 3.67 Å). Typical values for the sum of covalent radii are 2.07 Å for Re⋯N, 2.03 Å for Re⋯O and 2.74 Å for Re⋯Re. Similarly at Asp52*B* rhenium atom coordination occurs [Re–OD2 = 2.1 (2) Å; occupancy of 26%] and at Asn46*B* [Re⋯OD1 = 3.9 (2) Å and Re⋯ND2 = 4.7 (2) Å]. Lastly, coordination also occurs at Asp119*B* [Re–OD2 = 2.3 (2) Å, Re–OD1 = 3.1 (2) Å; occupancy of 62%].

**Figure 3:**
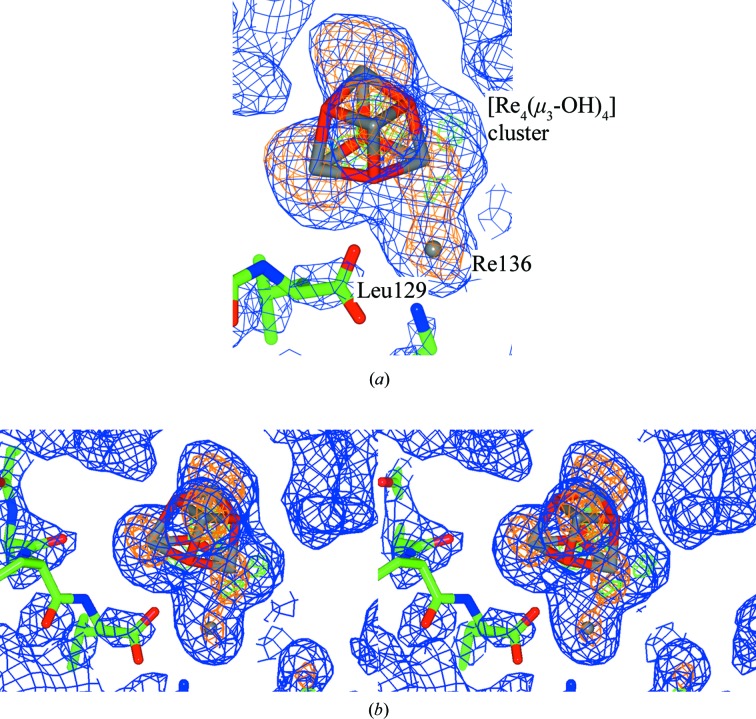
Complex rhenium tetranuclear species [Re_4_(μ_3_-OH)_4_(CO)_12_] at Leu129*A*, shown as a standard (top) and as a stereo image (bottom). A monomer CIF ligand QEB that was utilized as minimal density is present for the CO ligands and, therefore, they have been omitted. Blue shows the 2*F*_o_ − *F*_c_ electron-density map contoured at 1.2 r.m.s., green shows the *F*_o_ − *F*_c_ electron-density map contoured at 5.0σ (the *COOT* default; Emsley & Cowtan, 2004 ▸) and orange shows the anomalous electron-density map contoured at 3.0σ. This figure was prepared using *CCP4mg* (McNicholas *et al.*, 2011 ▸). The weak anomalous peak at the bottom position for a rhenium is indicative of this being an intermediate at the 1 year time point.

A complex cluster type is formed at Leu129*A* (Fig. 3 ▸), involving a mononuclear rhenium atom (Re136*B*; occupancy 39%) and a defined tetranuclear rhenium cluster indicated by the four Re apex and μ-OH atoms (occupancy 50%).

A non-bonded rhenium atom occurs in the vicinity of Leu75*B*, Trp63*B* and Asp101*B* (occupancy 25%), as well as at Ala107*B* (occupancy of 25%) and Gly71*B* (occupancy of 15%)

Tetranuclear rhenium clusters, with significant rhenium anomalous peaks and 2*F*_o_ − *F*_c_ density to allow the identification of the rhenium atoms, occur at six positions within the protein, namely at Leu129*A* (occupancy 50%; as specified above); Arg5*A* (occupancy 45%); Trp63*A*–Ser100*A* (occupancy 42%); Gly71*A*–Trp62*A*–Arg61*A* (occupancy 45%); Pro70*A* (occupancy of 27%); and at Asn103*A*–Arg112*A* (occupancy of 13%). Insufficient 2*F*_o_ − *F*_c_ density is available to fully refine the carbonyl ligands and these have been removed from the refinement utilizing the monomer CIF ligand, QEB.

**Figure 4:**
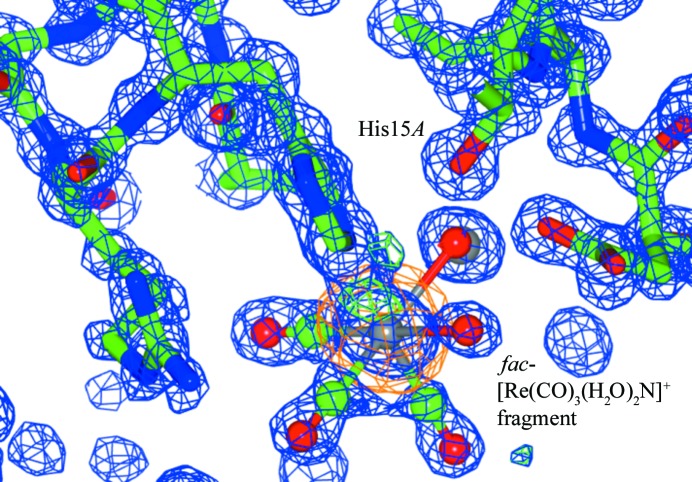
Rhenium mononuclear coordination *fac*-[Re(CO)_3_(H_2_O)_2_N] (N = His15) to His15*A* binding site for the 2Yr-X structure. Blue is the2*F*_o_ − *F*_c_ electron density map contoured at 1.2 r.m.s.; green is the *F*_o_ − *F*_c_ electron density map contoured at 5.0σ (the *COOT* default (Emsley & Cowtan, 2004 ▸); orange is the anomalous electron density map contoured at 3.0σ. This figure was prepared using *CCP4mg* (McNicholas *et al.*, 2011 ▸).

The two-year chemical-exposed structure (2Yr-X) crystallized in a tetragonal space group (*P*4_3_2_1_2; resolution = 1.03 Å) and shows rhenium coordination at the His15 residue with a bond distance of 2.16 (3) Å and an occupancy of 75% (Fig. 4 ▸). The bond distance is within the range of related small molecule crystallographic data of *fac*-[Re(CO)_3_(N_imidazole_)] 2.174 (4)–2.197 (5) Å (Schibli *et al.*, 2000 ▸; Garcia *et al.*, 2000 ▸; Fernández-Moreira, *et al.*, 2014 ▸; Brink *et al.*, 2013*a* ▸). The octahedral environment of the *fac*-[Re(CO)_3_(H_2_O)_2_N]^+^ is clearly visible and has been refined with the monomer CIF ligand RRE. Unlike previous reports, electron density, in addition to what is expected for an aqua ligand, is clearly visible with an Re—*X* bond distance of 2.61 (3) Å. The Re—Br bond can readily be substituted by H_2_O or a solvent molecule as indicated by kinetic studies (Alberto *et al.*, 1999 ▸; Schutte *et al.*, 2011 ▸; Brink *et al.*, 2013*b* ▸), and therefore has been refined as H_2_O/Br positional disorder. Small molecule Re—Br bond distances typically range from 2.60 to 2.65 (1) Å (see Fig. S1) (CSD version update 5.39, utilizing *Mogul*; Bruno *et al.*, 2004 ▸). The occupancy of the position disorder of the Br atoms is 35%.

**Figure 5:**
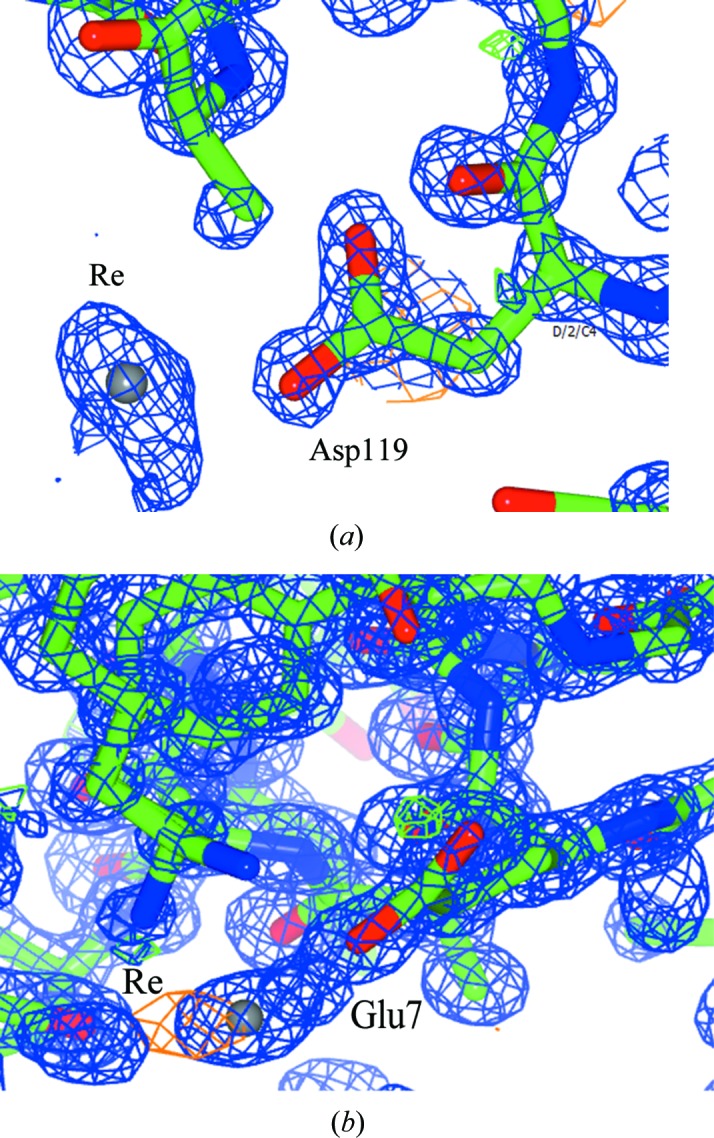
Rhenium coordination to (*a*) Asp119 and (*b*) Glu7. Blue shows the 2*F*_o_ − *F*_c_ electron-density map contoured at 1.2 r.m.s., green shows the *F*_o_ − *F*_c_ electron-density map contoured at 5.0σ (the *COOT* default; Emsley & Cowtan, 2004 ▸) and orange shows the anomalous electron-density map contoured at 3.0σ. This figure was prepared using *CCP4mg *(McNicholas *et al.*, 2011 ▸).

The 2Yr-X structure shows a single rhenium in the vicinity of Asp119 [Re–OD2 = 2.27 (4) Å and Re⋯OD1 = 3.52 (4) Å] with occupancy of 27%. Monodentate coordination occurs between rhenium and Glu7 [Re–OE2 = 2.07 (4) Å] with a rhenium occupancy of 15% (Fig. 5 ▸).

**Figure 6:**
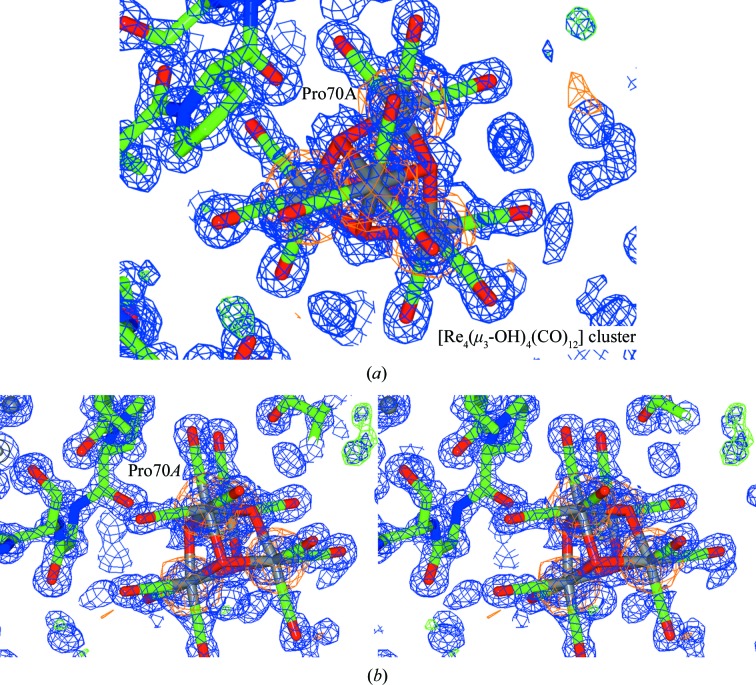
View of a standard (top) and a stereo image (bottom) of the well resolved rhenium tetranuclear cluster [Re_4_(μ_3_-OH)_4_(CO)_12_] in the vicinity of Pro70. Monomer CIF ligand KBW was utilized with definable 2*F*_o_ − *F*_c_ density for the CO ligands. Blue shows the 2*F*_o_ − *F*_c_ electron-density map contoured at 1.2 r.m.s., green shows the *F*_o_ − *F*_c_ electron-density map contoured at 5.0σ (the *COOT* default; Emsley & Cowtan, 2004 ▸) and orange shows the anomalous electron-density map contoured at 3.0σ. This figure was prepared using *CCP4mg* (McNicholas *et al.*, 2011 ▸).

A well resolved rhenium tetranuclear cluster [Re_4_(μ_3_-OH)_4_(CO)_12_] (occupancy of 75%), occurs in the vicinity of Pro70 with a Pro70 O⋯μ-OH interaction of 2.58 (2) Å, within the range of typical O⋯O van der Waals interactions (3.04 Å). The tetranuclear cluster shows significant anomalous density (19.8σ) for the four rhenium apex atoms and clearly defined 2*F*_o_ − *F*_c_ density for the hydroxide and carbonyl ligands (Fig. 6 ▸).

A second cluster occurs in the vicinity of Arg5, Trp123 and Lys33, with an occupancy of 25% and anomalous density of 6.9σ. Only the positions of the rhenium atoms are clearly defined with little to no density for the 12 carbonyl ligands (indicated for the purpose of chemical accuracy with zero occupancy), despite an initial *F*_o_ − *F*_c_ density of 5σ with the preliminary placement of the rhenium atoms.

A *fac*-[Re(CO)_3_(H_2_O)_3_]^+^ monomer (occupancy 22%) occurs within close proximity to the second cluster [Re⋯Re 5.53 (2) Å; Re⋯μ-OH 4.97 (2) Å] with partial 2*F*_o_ − *F*_c_ density for the carbonyl ligands. A third cluster occurs at Gly117 with an occupancy of 27% with the rhenium atoms defined by the anomalous 2*F*_o_ − *F*_c_ density.

## 4. Conclusions   

In the 1Yr-Y crystal structure, it is remarkable that these clusters form by drawing to them rheniums bound to different amino acids, all except the tri­carbonyl rhenium which remains bound to the histidine. Of course, it is not understood how the solvent and solutes involved in the protein crystallization, and the crystal mother liquor in the open solvent channels of the crystal, influence rhenium cluster formation. However, from literature reports we know that time and absolute metal concentration is a factor in cluster formation as well as the presence of organic solvents such as DMSO, used here in the initial dissolution of the rhenium tri­carbonyl tri­bromo compound.

In the 2Yr-X crystal structure, it is reassuring that the clusters are generally stable. Indeed the tetrarhenium cluster formed under both acidic and basic conditions and is known to be chemically very stable. This is a good property for an *in vivo* application such as medical imaging, where the compound would obviously be administered to a patient as an already synthesized rhenium cluster.

These time-resolved protein crystallography results also reveal occupancy variations in some of the rheniums, as well as stable clusters of others, while ending with a completed, very well resolved, tetrarhenium cluster after two years.

One can combine these protein crystallography results with the recent chemical crystallography results (Mokolokolo *et al.*, 2018 ▸) of the formation of mixed Re_3_Tc_1_ tetranuclear clusters for application in theranostic radiopharmaceuticals, which allows the introduction of a dual pharmaceutical – one with both imaging and therapy purposes. Therefore, this research confirms that loading a patient with high concentrations of the mononuclear rhenium, or with a pre-formed tetranuclear cluster, suggests suitability for medical diagnosis because of its stability, preference of formation and biological compatibility.

## Supplementary Material

Supplementary table and figures. DOI: 10.1107/S2052252519006651/lz5026sup1.pdf

PDB reference: 1Yr-Y, lysozyme with Re cluster, one year on the shelf, 6ro5

PDB reference: 2Yr-X, lysozyme with Re cluster, two years on the shelf, 6ro3

Raw diffraction images. Formation of a highly dense tetra rhenium cluster in a protein crystal and its implications in medical imaging.: https://doi.org/10.5281/zenodo.2874342

## Figures and Tables

**Table 1:** X-ray crystallographic data and final protein model refinement statistics for the one-year on-the-shelf (1Yr-Y) and two-year on-the-shelf (2Yr-X) structures in comparison to the freshly crystallized structure Overall diffraction resolution values are given, with the outer diffraction resolution shell values given in parentheses.

	Brink & Helliwell (2017 ▸)	1Yr-Y (6ro5)	2Yr-X (6ro3)
Data reduction			
Space group	*P*2_1_2_1_2_1_	*P*2_1_2_1_2_1_	*P*4_3_2_1_2
Unit-cell parameters (Å, °)	*a* = 36.98 (3), *b* = 79.80 (1), *c* = 79.92 (1), α = β = γ = 90	*a* = 37.88 (3), *b* = 78.65 (1), *c* = 80.67 (1), α = β = γ = 90	*a* = 79.94 (1), *b* = 79.94 (1), *c* = 36.46 (3), α = β = γ = 90
Molecular mass (Da)	14700	14700	14700
Molecules per asymmetric unit	2	2	1
Detector	Dectris PILATUS 6M-F	Dectris PILATUS 6M-F	Dectris PILATUS 6M-F
Crystal-to-detector distance (mm)	135	135	135
X-ray wavelength (Å)	0.97625	0.9763	0.9763
Observed reflections	735464 (99591)	184281 (7868)	108682 (6359)
Unique reflections	63838 (9126)	29804 (1421)	55335 (3732)
Resolution (Å)	56.47–1.26	31.43–1.68 (1.74–1.68)	39.97–1.03 (1.067–1.03)
Completeness (%)	99.9 (99.5)	99.86 (99.82)	94.04 (64.31)
*R*_merge_	0.077 (1.453)	0.06629 (0.5488)	0.09022 (0.1646)
〈*I*/σ(*I*)〉	14.7 (1.6)	14.6 (1.2)	38.7 (3.3)
Multiplicity	11.5 (10.9)	6.2 (5.5)	11.9 (10.4)
Mn(*I*) half-set correlation CC_1/2_	0.998 (0.536)	0.991 (0.642)	0.998 (0.897)
Cruickshank DPI (Å)	0.050	0.129	0.018
Average *B* factor (Å^2^)	22.8	37.06	10.64
Refinement			
*R* factor/*R*_free_ (%)	17.9/22.6	24.07/27.19	11.11/11.78
*R* factor overall (%)	18.2	24.25	11.21
R.m.s.d. angles (°)	2.793	0.99	1.36
Ramachandran plot values (%)			
Most favoured	96.6	97.64	98.43
Additional allowed	3.44	2.36	1.57
Disallowed	0	0	0
