# Supplementary material for: Formation of a highly dense tetra-rhenium cluster in a protein crystal and its implications in medical imaging
Source: IUCrJ. 2019 Jun 13;6(Pt 4):695–702. doi: 10.1107/S2052252519006651 (PMC6608631; doi:10.1107/S2052252519006651)
Supplement: Supplementary file 1 [file m-06-00695-sup1.pdf]

# IUCrJ

**Volume 6 (2019)**


**Supporting information for article:**

## Formation of a highly dense tetra-rhenium cluster in a protein crystal and its implications in medical imaging

**Alice Brink and John R. Helliwell**

**Table S1** Summary of all rhenium binding in three structures after crystallizations, and time on shelf collections at 1 and 2 years.

| **IUCrJ 2017**<br>**Fresh crystallised** | **1Yr –Y** | **2Yr – X** |
|---|---|---|
| Residue<br>(Orthorhombic $P2_12_12_1$) | (Orthorhombic $P2_12_12_1$) | (Tetragonal $P4_32_12$) |
| His 15 A & B | His 15 A & B | His 15 |
| | | |
| Asp 119A | Asp 119 A & B | Asp 119 |
| Asp 18 A & B | Asp 18A | |
| Asp 52 A & B | Asp 52 A & B | |
| | | |
| Glu 7B | Glu 7A (non bonded) | Glu 7 |
| Glu 35 A &B | Glu 35 A | |
| | | |
| Arg 125 B | Arg 125A | |
| Arg 61B | | |
| | | |
| Leu 125 A ?? | | |
| Leu 129 B | Leu 129B | |
| | | |
| Non-bonded in vicinity of | Non-bonded in vicinity of | Non-bonded in vicinity of |
| Leu 129 B | Leu 129A | |
| Pro 70 A and Arg 61A | | |
| Arg 14 B | | |
| | Glu 7A and Lys 1A | |
| | Trp 63B | |
| | Asp 101B | |

| | | |
|---|---|---|
| | Ala 107B | |
| | Gly 71 B | |
| Cluster Formation | Cluster Formation | Cluster Formation |
| | Leu 129 A | |
| | Arg 3A | |
| | | Arg 5 |
| | Trp63 A / Ser 100A | |
| | | Trp 123 |
| | Pro 70A | Pro 70 |
| | | |
| | Asn 103 | |
| | | Lys 33 |
| | | |

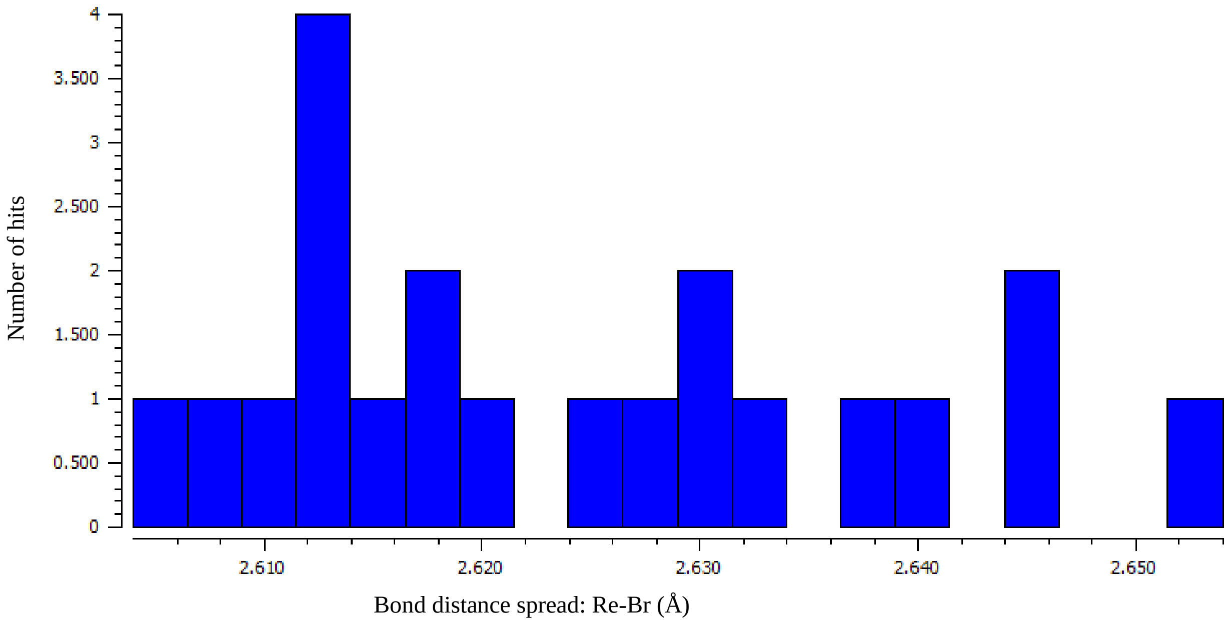


**Figure S1** Comparison of all possible small molecule Re-Br bond distances. *Mogul* (Bruno et al., 2004)[1], CSD Data analysis (version update 5.39) of Re-Br bond distances of small molecule hits utilising the Re-Br distance found within the *fac*-$[Re(CO)_3(N)(O)]^+$ fragment as search criteria found on the CSD database, with a total of 21 Hits. Colour bars indicate number of structure hit entries with respect to bond length (Å) in data libraries used: CSD version update 5.39. Minimum/maximum bond distances (2.60 / 2.65 Å) with a standard deviation of 0.014 Å and a mean value of 2.62 Å.

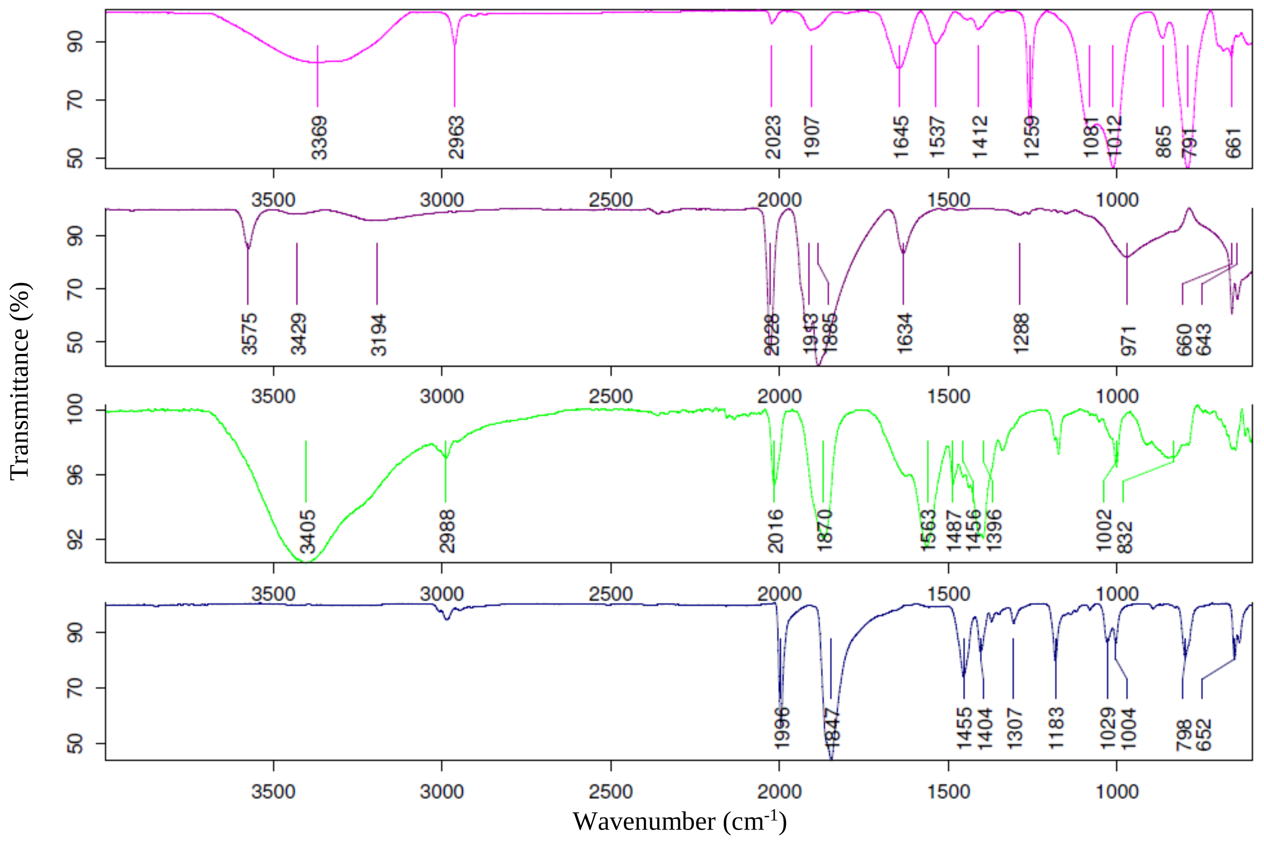


**Figure S2** ATR-IR spectra of the rhenium complexes. (A) IR of crystals contain HEWL-rhenium with cluster formation. Crystals remained for two years in sitting drop in the specified buffer solution. (B) Solid state IR of $[Re_4(\mu_3\text{-}OH)_4(CO)_{12}]$ cluster complex, synthesised according to Egli, et al., 1997. (C) Solid state IR of *fac*-$[Re(CO)_3(Br)_3]^{2+}$ dissoved in 10% NaCl, NaOAc 0.04 M (pH 4.7) for 3 months. (D) Solid state IR of *fac*-$[Re(CO)_3(Br)_3]^{2+}$.

---

[1] Bruno, I. J., Cole, J. C., Kessler, M., Luo, J., Motherwell, W. D. S., Purkis, L. H., Smith, B. R., Taylor, R., Cooper, R. I., Harris, S. E. & Orpen, A. G. (2004). *J. Chem. Inf. Comput. Sci*. **44**, 2133–2144.
